# Diets of Three Sympatric Grebe Species in Lake Titicaca Indicate Possible Dietary Niche Partitioning

**DOI:** 10.1002/ece3.72123

**Published:** 2025-09-29

**Authors:** D. A. Villar, Ever Yanes, Edwin R. Gutiérrez Tito, Andrew G. Gosler

**Affiliations:** ^1^ Edward Grey Institute of Field Ornithology, Department of Biology University of Oxford Oxford UK; ^2^ Department of Anthropology Durham University Durham UK; ^3^ Facultad de Ciencias Biológicas Universidad Nacional del Altiplano Puno Peru; ^4^ Parque Nacional Bahuaja‐Sonene Servicio Nacional de Áreas Naturales Protegidas por el Estado Puerto Maldonado Peru; ^5^ Institute of Human Sciences University of Oxford Oxford UK

## Abstract

Dietary niche partitioning is a fundamental process in community ecology, of interest to both conservationists and ecologists. We present data on the dietary niches of three sympatric grebes from Lake Titicaca: the endangered endemic Titicaca Grebe *Rollandia microptera* , Rolland's Grebe *Rollandia rolland* , and the Silvery Grebe *Podiceps occipitalis* . This is the first study comparing the diets of these three species since 1981. We conducted stomach content analysis on 45 Titicaca Grebes, 30 Rolland's Grebes, and 15 Silvery Grebes, and quantified the degree of niche overlap among individuals and what prey classes were driving variation between species. Rolland's Grebe and the Silvery Grebe have a more invertebrate‐based diet than the Titicaca Grebe. The use of presence‐absence methodologies and numeric methodologies for counting prey items leads to different results regarding dietary differences between these species. While our dietary categories are too coarse to definitively prove it is a case of dietary niche partitioning, it is indicative of it. We suggest future avenues to prove dietary niche partitioning in these species, and how dietary studies could answer questions regarding this ecosystem's response to invasive species.

## Graphical abstract

Lake Titicaca is home to three grebe species whose diets have not been thoroughly studied. In this natural history note, we provide the first description of the diets of these species and how they overlap and differentiate.

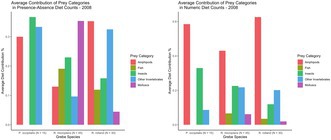

## 1 Introduction

While it is common for similar species to coexist across a variety of taxa, classical ecological theory suggests that it is not possible for two species to coexist if they have completely overlapping ecological niches (Gause 1932; Lack 1946; Hardin 1960). Niche partitioning is the process by which species that can potentially compete for the same resource change their behavior, either through adaptive evolution or behavioral plasticity, to reduce the overlap of multiple species niches within a given area to the degree that permits the coexistence of these species (MacArthur and Levins 1967). This process of niche partitioning is fundamental to community ecology and is often used to explain how sympatric species coexist (Schoener 1974). Across a variety of taxa, including birds, diet is often used as a measure of niche partitioning between species (Lack 1946; Fjeldså 1981, 1983; Gosler 1990; Gosler and Carruthers 1994). For instance, Fjeldså (1983) analyzed the dietary overlap of grebes in Europe, South America, and Oceania, and found that where grebe species are sympatric, the bill morphology changes between them to lessen the dietary overlap between them. He related this both to the available prey and to the foraging patterns of the species in question.

Three grebe species live in Lake Titicaca: the Titicaca Grebe ( *Rollandia microptera* ), Rolland's Grebe ( *Rollandia rolland* ) and the Silvery Grebe ( *Podiceps occipitalis* ). The Titicaca Grebe is a flightless endemic found only in Lake Titicaca and connected bodies of water (Martinez et al. 2006; Villar et al. 2023). It nests in mosaics of open water, weighs up to 600 g, and is primarily piscivorous (Fjeldså 2004). It is classified as endangered due to rapid population declines in the late 20th and early 21st centuries (Martinez et al. 2006), but there are indications that it has recovered slightly from an early 21st nadir (Villar, Velásquez‐Noriega, et al. 2024). Rolland's Grebe weighs between 100 and 150 g, and while not flightless like the Titicaca Grebe, it is not capable of long‐distance flights. It primarily nests in *llacho* vegetation and is thought to primarily eat small fish and arthropods (Fjeldså 2004). The Silvery Grebe has a weight intermediate between the Titicaca Grebe and the Rolland's Grebe, usually around 300 to 400 g. It is considered an insectivore, primarily eating aquatic invertebrates. Unlike the highly territorial Titicaca and Rolland's Grebes, the Silvery Grebe nests near conspecifics, usually on mats of floating vegetation (Ibid.).

The grebes of the Lake Titicaca region have been subject to one extensive comparative ecological study, which included an analysis of prey species of the grebe species of Lake Titicaca (Fjeldså 1981). The section on grebe diets in this monograph includes raw data, but the focus was on the effect of bill morphology on dietary niche partitioning. It found that bill morphology had a significant effect on diet within species and suggested that this, rather than species, was the primary method of dietary niche partitioning in Lake Titicaca. However, it did note that there was a degree of species partitioning, with the Silvery Grebe particularly having a diet heavier in cladocerans and the Titicaca Grebe having a diet with more fish. However, this partitioning could also be due to geographic differences in the distribution of the three species, as the Silvery Grebe was not found in Lake Titicaca itself but rather found in outlying lakes of the Altiplano region. It could also be due to habitat differences between the species, as the Titicaca Grebe forages in more open water than Rolland's Grebe or the Silvery Grebe (Fjeldså 2004).

The purpose of this study is to assess potential niche partitioning among the three grebe species of Lake Titicaca using stomach content analysis. We make no formal attempt to compare our dietary results with those of the 1981 study, both because of the differences in sample size and because of the uncertainty in comparing stomach contents results obtained by different people using different methods, decades apart.

## 2 Methods

### 2.1 Study Sites

Lake Titicaca is, at 3814 m.a.s.l., the world's highest altitude navigable lake (Wirrmann 1992). The lake covers 8299 km^2^ across Peru and Bolivia and includes a variety of environments, including a large expanse of totora (*Schoenoplectus californcius tatora*) sedge wetlands in Puno Bay. Most of these wetlands are under the protection of the Reserva Nacional del Titicaca, the only protected area in Lake Titicaca, which was created in 1978 and has been administered by the Servicio Nacional de Áreas Naturales Protegidas por el Estado (SERNANP).

Lake Titicaca is a globally unique high‐altitude freshwater and wetland ecosystem, which has been declared to be an Important Bird Area by BirdLife International and a RAMSAR wetland (RAMSAR 2023; BirdLife International 2024). It is home to, including vagrants and seasonal migrants, an estimated 135 bird species (Pulido Capurro 2018), including the endangered endemic Titicaca Grebe (Martinez et al. 2006; Villar et al. 2023). It is also an important habitat for two other grebe species, the Silvery Grebe and Rolland's Grebe (Fjeldså 2004). However, the ecosystem is experiencing significant threats. Invasive rainbow trout (*Salmo gairdneri*) and pejerrey, also known as silverside in English ( *Odontesthes bonariensis* ), introduced in the mid‐20th century (Everett 1973), are associated with dramatic changes in fish and invertebrate community assemblages (Monroy et al. 2014) and with the extinction of the Titicaca Orestias (*Orestias cuvierii*) (Vila et al. 2007). The associated fisheries of introduced and native fish are a major threat to the endemic fish (Vila et al. 2007) through overfishing and to birds, including the Titicaca Grebe, which often get caught as bycatch (Martinez et al. 2006; Quispe et al. 2023; Villar, Thomsen, et al. 2024; Villar, Yanes, et al. 2024; Villar et al. 2025). There are also risks from pollution, both from untreated human wastewater (Archundia et al. 2017) and from mining around the Lake Titicaca watershed (Cáceres Choque et al. 2013). More recently, climate change has caused significant declines in the water level of Lake Titicaca, with significant cascading ecological and social consequences (Zubieta et al. 2021).

**FIGURE 1:**
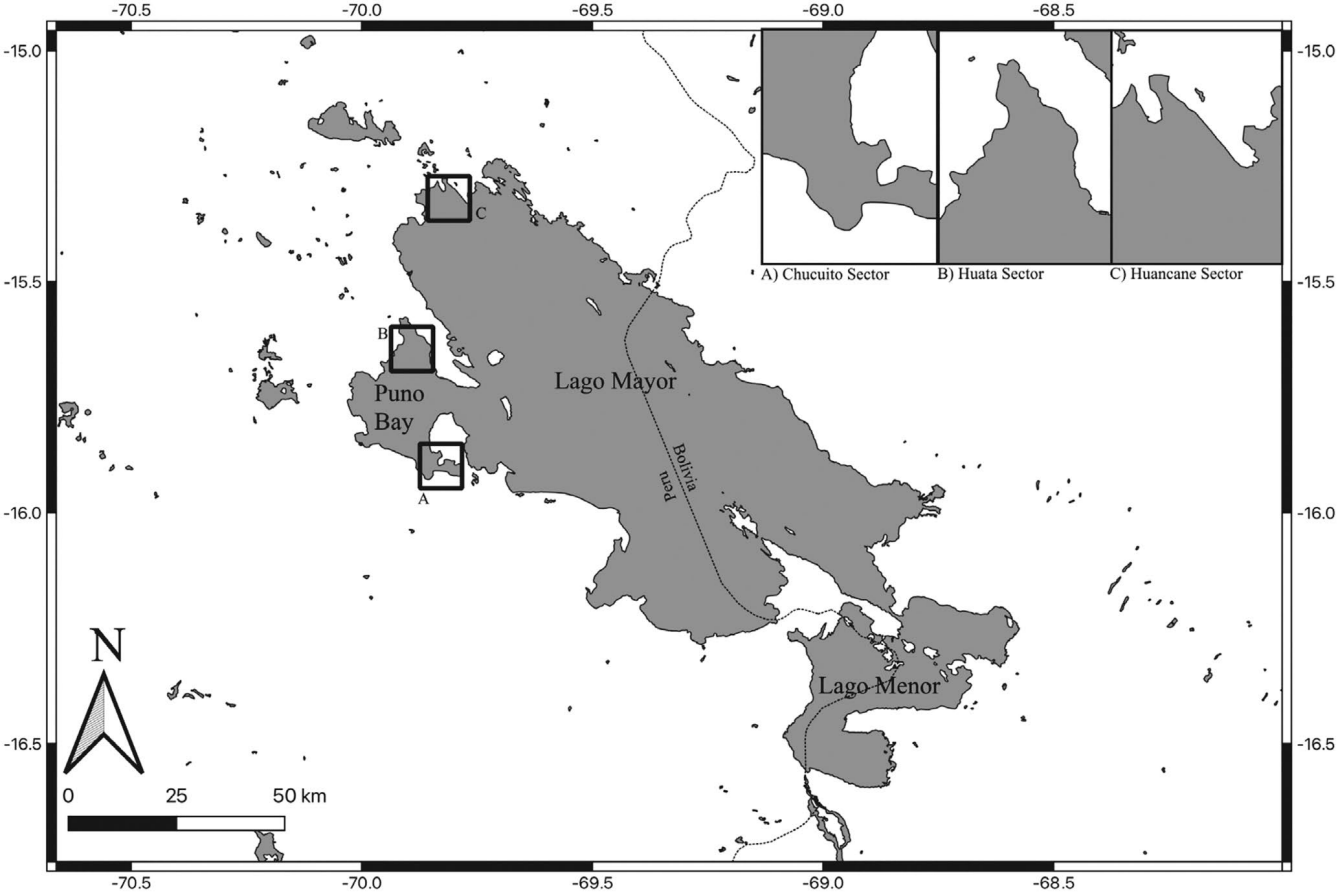
Map of Lake Titicaca, with the Huata, Chucuito, and Huancané Sectors where birds were collected in insets.

Grebe specimens were collected from three sites on the Peruvian side of Lake Titicaca: Huancané, Huata, and Chucuito (Figure 1). Huancané is characterized by deep waters, going over 10 m in parts, and little vegetation. What vegetation exists tends to be surface algae. Fishing is common in this region. Huata is in Puno Bay and near the Reserva Nacional del Titicaca. It has shallow (usually under 3 m) waters and is primarily covered by totora sedge wetlands. These totora wetlands mean that you get less intense fishing in this region, though hunting, both with shotguns and snares, is common. Chucuito is in the south of Puno Bay and has shallow water like Huata but also has limited aquatic vegetation like Huancané. Fishing is common in this region, though at the time of the collection of these specimens, fish farming was beginning to supplant fishing as the primary economic use of the water in this sector. To avoid model overfitting, we have grouped individuals from different sites.

### 2.2 Specimen Collection and Stomach Content Analysis

Grebe specimens were collected between February and September, 2008. Individuals were collected by local fishers when found dead as bycatch in their monofilament nets. Individuals were then placed in 40% ethanol and transported overland to the Universidad Nacional del Altiplano. Stomach content analysis was performed on the same day as the specimens' arrival at the Universidad Nacional del Altiplano.

The stomach contents were removed from each bird via an incision on its ventral side. Stomach content was then identified by reference to a voucher collection, with fish being identified by otolith and invertebrates identified by exoskeleton and shell remnants. Due to both limitations in the voucher collection used and the difficulties in identifying semi‐digested fish remains to the level of species, we identified fish solely to the level of genus. To not inflate the importance of individual invertebrate species and due to similar issues in identifying partially digested samples, invertebrates were grouped into broader taxonomic groups, leading to a total of five dietary categories: fish, insects, amphipods, molluscs, and other invertebrates.

There are a variety of methods to analyze stomach contents, including volumetric, presence‐absence, and numeric (Amundsen and Sánchez‐Hernández 2019; Hyslop 1980). Due to the difficulties in establishing the volume of prey in stomach contents analysis accurately (Rindorf and Lewy 2004), we opted to not include volumetric measures in our analysis. There is a significant body of literature, primarily from fisheries science, focusing on the benefits and drawbacks of various methods of stomach content analysis (Amundsen and Sánchez‐Hernández 2019). Numerical methods have the benefit of allowing the relative importance of different prey to be measured (Hynes 1950), but have a tendency to overstate the importance of small, numerically plentiful, but energetically negligible prey species (Ahlbeck et al. 2012). Presence–absence methods do not permit us to assess the relative importance of different prey species within the diet of individuals, which could lead to misleading dietary interpretations in cases where one or a few diet classes dominate across members of a species. This can be an issue for studying the diet of dietary specialists, but presence‐absence methods have the advantage of providing a dataset that is more mathematically robust (Baker et al. 2014; Buckland et al. 2017). Following (Amundsen and Sánchez‐Hernández 2019) we included both numeric and presence‐absence counts in our analysis of the diet of the three sympatric grebes in this study.

### 2.3 Statistical Analysis

Data was square root transformed to reduce the influence of skewed data (Platell and Potter 2001). We constructed Bray–Curtis dissimilarity matrices from this transformed data (Bray and Curtis 1957) in R for both numeric and presence‐absence prey counts. The Bray–Curtis dissimilarity matrices were used for conducting Analysis of Similarity (ANOSIM) (Clarke 1993), with 9999 permutations, to study whether there were statistically significant differences between species, using the *anosim()* function from the *vegan* R package (Oksanen et al. 2001). The results of the ANOSIM were then evaluated using the ANOSIM statistic *R*, which runs from −1 to 1. We used Similarity Percentages (SIMPER) analysis (Clarke 1993) to assess the contribution of individual prey categories to differences between species, using the *simper()* function of the *vegan* R package. Following previous studies (e.g., Baje et al. 2022), we assessed which prey categories were significant for pairwise dissimilarity by ranking the contribution to dissimilarity in descending order, including all prey categories that contributed to at least 70% of the pairwise dissimilarity.

In addition to these analyses, we used Schoener's Overlap Index, usually represented as *D* (Schoener 1970), to calculate pairwise comparisons in diet composition between species. This was done with non‐square root transformed data for both presence‐absence and numeric counts. Schoener's *D* is calculated as $D=1-0.5\;\left(\sum_{i=1}^{n}\left|p_{\mathrm{ij}}-p_{\mathrm{ik}}\right|\right)$, where *p*_*ij*_ is the proportion of prey type *i* in the diet of population *j*, and *p*_*ik*_ is the proportion of prey type *i* in the diet of population *k*. The span of the index is from 0, indicating no overlap, and 1, indicating complete overlap. A value of over 0.6 is a biologically significant overlap in dietary niche (Wallace 1981). Schoener's *D* was calculated using the *dietOverlap()* function of the *FSA* R package (Ogle et al. 2015).

All analyses were done in R version 4.4.0, with figures being made in R using the ggplot2 package (Wickham 2016) and QGIS. Raw data and R code used for this paper can be found in the Supporting Information.

## 3 Results

We studied the stomach contents of 15 Silvery Grebes, 30 Rolland's Grebes, and 45 Titicaca Grebes. We found 14 adult and 1 juvenile Silvery Grebes, 11 adult and 4 juvenile Rolland's Grebes, and 12 adult and 3 juvenile Titicaca Grebes in the Huata Sector, 9 adult and 6 juvenile Rolland's Grebes and 5 adult and 10 juvenile Titicaca Grebes in the Chucuito Sector, and 9 adult and 6 juvenile Titicaca Grebes in the Huancané Sector.

**FIGURE 2:**
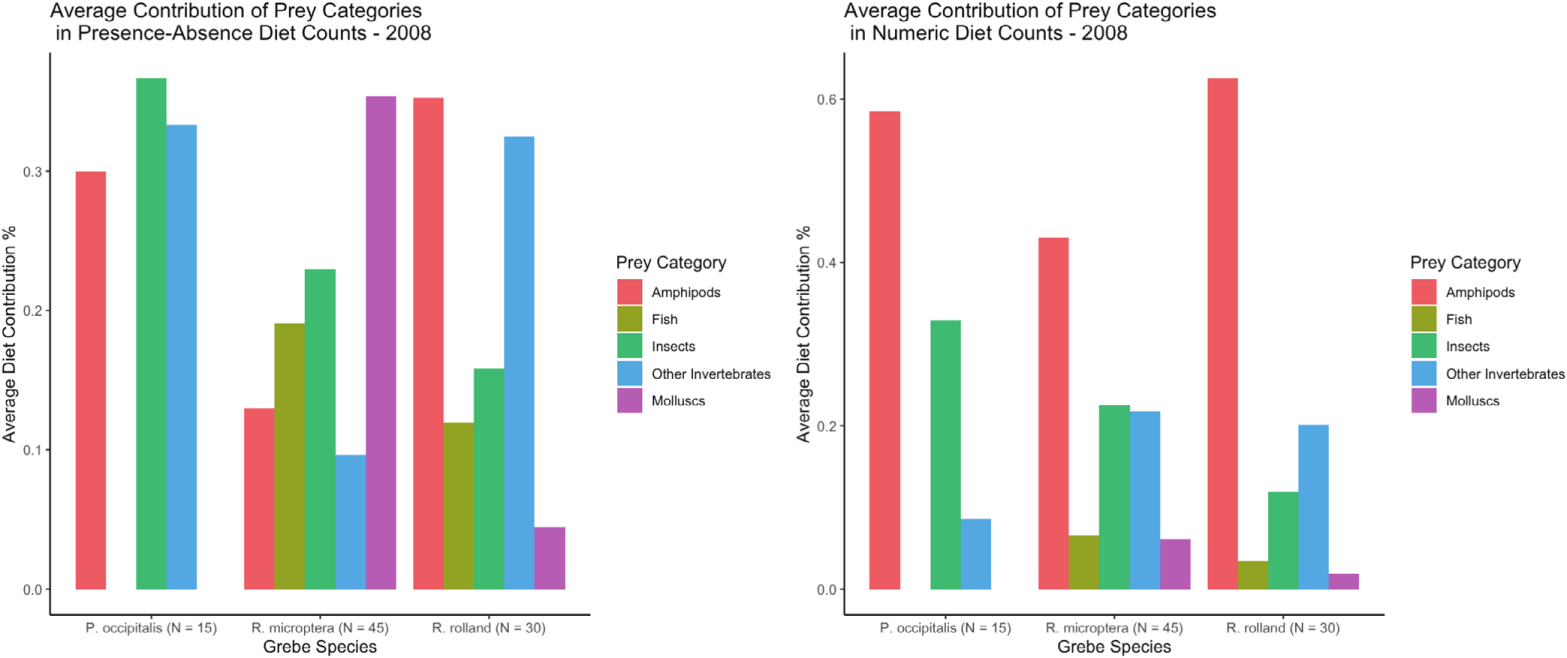
Average contribution of prey categories to species diets.

Amphipods were the largest prey category for all three species, contributing an average of 58.4% (SD = 29.9) of prey items found in the stomach of Silvery Grebes, 43.0% (SD = 36.6) of prey items found in the stomach of Titicaca Grebes, and 62.6% (SD = 25.3) of prey items found in Rolland's Grebes in the numeric dietary counts. However, the grebe species differed in which prey categories were the next most important in contributing to their diet: for Silvery Grebes, molluscs and insects formed the bulk of their non‐amphipod diet, whereas Titicaca Grebes consumed on average nearly equal amounts of insects and molluscs, followed by fish and other invertebrates, and Rolland's Grebe averaged larger numbers of molluscs and insects in their diet (Figure 2). Using the presence‐absence counts, we found that insects, amphipods, and molluscs all contributed nearly equally to the diet of Silvery Grebes, while the average contribution of other invertebrates and fish increased in the diet of the Titicaca Grebe, and the average contribution of fish and insects increased in the diet of Rolland's Grebe (Figure 2).

The ANOSIM found a significant difference between the numeric diet counts of the three grebe species (*R* = 0.059, *p* = 0.049), but it found no statistically significant difference in the presence‐absence of prey items in diet between the species (*R* = −0.040, *p* = 0.899). SIMPER found that, in descending order, amphipods, insects, and molluscs were the largest drivers of dissimilarity between the numeric diet counts of the Titicaca Grebe and Rolland's Grebe. It found that amphipods and insects were the largest drivers of dissimilarity between the numeric diet counts of the Titicaca Grebe and the Silvery Grebe using the numeric count. It found that amphipods and insects were the largest drivers of dissimilarity between Rolland's Grebe and the Silvery Grebe using the numeric count (Table 1). SIMPER found that, in descending order, insects, fish, amphipods, and molluscs were the largest drivers of dissimilarity between the presence‐absence of prey items in the diets of the Titicaca Grebe and Rolland's Grebe and between the presence‐absence of prey items in diet of the Titicaca's Grebe and the Silvery Grebe. Insects, other invertebrates, and fish were the largest drivers of dissimilarity of the presence‐absence of prey items in diet between the Rolland's Grebe and the Silvery Grebe (Table 2).

**TABLE 1:** Contribution of prey categories to pairwise dissimilarity of numeric diets, with cumulative percentage contributions in brackets.

Comparison	Prey 1	Prey 2	Prey 3	Prey 4	Prey 5
Titicaca Grebe and Rolland's Grebe	Amphipods (0.368)	Insects (0.610)	Molluscs (0.804)	Fish (0.906)	Other invertebrates (1.00)
Titicaca Grebe and Silvery Grebe	Amphipods (0.444)	Insects (0.765)	Molluscs (0.891)	Fish (0.949)	Other invertebrates (1.00)
Rolland's Grebe and Silvery Grebe	Amphipods (0.425)	Insects (0.793)	Molluscs (0.898)	Other invertebrates (0.961)	Fish (1.00)

**TABLE 2:** Contribution of prey categories to pairwise dissimilarity of presence‐absence of prey items, with cumulative percentage contributions in brackets.

Comparison	Prey 1	Prey 2	Prey 3	Prey 4	Prey 5
Titicaca Grebe and Rolland's Grebe	Insects (0.235)	Fish (0.465)	Amphipods (0.646)	Molluscs (0.825)	Other invertebrates (1.00)
Titicaca Grebe and Silvery Grebe	Insects (0.254)	Fish (0.484)	Amphipods (0.678)	Molluscs (0.852)	Other invertebrates (1.00)
Rolland's Grebe and Silvery Grebe	Insects (0.332)	Other invertebrates (0.550)	Fish (0.750)	Amphipods (0.880)	Molluscs (1.00)

The Schoener's Overlap index of numeric diet counts between the Silvery Grebe and the Titicaca Grebe (*D* = 0.742), the Silvery Grebe and Rolland's Grebe (*D* = 0.789), and the Titicaca Grebe and Rolland's Grebe (0.805) all indicate biologically significant dietary niche overlap. The Schoener's Overlap Index of the presence‐absence of prey items in diet also indicated biologically significant overlap between the diets of the Silvery Grebe and Rolland's Grebe (*D* = 0.783), but not between the Silvery Grebe and the Titicaca Grebe (*D* = 0.456) or between the Titicaca Grebe and Rolland's Grebe (*D* = 0.548).

## 4 Discussion

We found some indications of dietary niche partitioning between the three grebe species. Both presence‐absence and numeric counts suggested a degree of dietary niche partitioning, but which of these counts suggested it depended on the method used. We suspect that there is genuine dietary niche partitioning, but that it is likely being modulated by morphology that is not exclusively related to species. It is also likely that our dietary categories are too coarse to prove dietary niche partitioning, and some cryptic partitioning, especially regarding the types of invertebrates different species are targeting, is being lost. Previous work on grebes in Lake Titicaca suggests that bill morphology can impact dietary niche partitioning as much, if not more, than species (Fjeldså 1981), and that there is considerable intraspecific variation in bill morphology. In other systems, factors such as the sex of the individual in question (Bauld et al. 2022), the time at which foraging occurs (Rodriguez Curras et al. 2022), or the idiosyncratic prey preferences of individuals (Toscano et al. 2016) are important in niche partitioning between species. Follow‐up work on how bill morphology varies between species and how this relates to demographic factors such as age and sex could reveal how these grebe species coexist despite similar dietary niches. In addition to demographic factors, it is possible that geography or differences in habitat might be confounding our results. This is especially the case regarding the diet of the Silvery Grebe, which was not found in Lake Titicaca proper, but only in outlying bodies of water in 1981 (Fjeldså 1981). We are unsure when this species colonized Lake Titicaca, but it has been found in Lake Titicaca itself for at least twenty years (Author 2, pers. obvs.); this colonization might have influenced how different grebes partition food resources in the lake. We opted not to look at the effect of age or of site on diet because it would lead to model overfitting—however, further examination of the diet of these species could find that while dietary niches between the species across Lake Titicaca overlap considerably, they do not at individual sites.

While most previous research on the diet of the Silvery Grebe agrees that it primarily feeds on invertebrates (Hilsenbeck 1979; Fjeldså 1981), most previous work on both the Titicaca Grebe and Rolland's Grebe has described both species as primarily piscivorous (Fjeldså 1981). The perception by many local fishers of the Titicaca Grebe being a major competitor for fish is a major barrier to the conservation of this endangered species (Villar, Velásquez‐Noriega, et al. 2024), with previous conservation‐related dietary work on it focusing on the degree to which the fish targeted by the Titicaca Grebe and fishers overlapped (Villar, Thomsen, et al. 2024). Our results suggest that invertebrates are a significant part of the diet for all grebe species in Lake Titicaca, including the “piscivorous” Titicaca Grebe and Rolland's Grebe, and if there is dietary niche partitioning between these species, it is primarily modulated by invertebrate prey categories. Further work, with a finer taxonomic resolution of prey species, could reveal which species of invertebrates are most important in partitioning the diets of these grebe species. However, the importance of invertebrates in the diets of these grebes could be an artifact of the methodology used; previous work has suggested that while numerically dwarfed by invertebrate individuals, fish constitute more of the volume of the diet of both these species (Fjeldså 1981). The importance of invertebrates reflects changes in prey availability between the two studies; between the 1980s and the 2000s, fisheries expanded in Lake Titicaca, leading to significant overfishing (Villar 2025). Overfishing in Lake Titicaca might have changed the diet of usually piscivorous grebes towards invertebrates, akin to how overfishing of sardines and hake changed Stripped Dolphin ( *Stenella coeruleoalba* ) diets in the Mediterranean (Gómez‐Campos et al. 2011), and how overfishing of Argentine Hake ( *Merluccius hubbsi* ) led to the Yellownose skate (*Zearaja brevicaudata*) changing its diet to focus on smaller species (Tschopp et al. 2024).

Prey availability is fundamental to determining predator dietary niches (Fjeldså 1981). Unfortunately, information on the distribution of prey availability in Lake Titicaca for these species does not exist. The Peruvian Ministerio de la Producción, in conjunction with the Laboratorio Continental of the Instituto del Mar del Peru, collects data on fish landing from several fishing villages on the Peruvian side of Lake Titicaca that could act as a proxy for fish prey availability, similar to how limited fisheries catch records have been used to estimate fish abundance in data‐poor regions (Dowling et al. 2015). However, this analysis would require a larger sample size, which is not likely to be approved given ethical restrictions on lethal sampling, especially of endangered species. Even if such a larger sample size were obtained, such as through a multi‐year community conservation project with local fishers to collect their bycatch, there would still be no information on how invertebrate prey availability varies by site. This would have to be obtained by field surveys of invertebrate populations at different sites, and working with taxonomists to identify the invertebrate fauna of Lake Titicaca, much of which remains unknown to science (Villar 2025).

While this study offers an update on the diet of the grebes of Lake Titicaca, including how the Silvery Grebe has now moved to Lake Titicaca proper, it is still a nearly two‐decade‐old piece of data on grebe diets. Local conservationists will no doubt appreciate learning more about the diets of these species, especially of the endangered Titicaca Grebe, and can use this information to study how grebes and local fisheries are likely to interact. However, that does not negate the need for more recent studies of grebe diets, both to see how they changed in response to invasive salmonids in the 20th century, and how grebes partition the resources of Lake Titicaca between themselves, and between themselves and the local fisheries.

Stomach content analysis is one of the most widely used methods to study diets, and it is accurate. However, it is limited in that, by definition, the stomach contents are only the most recent meals of the individual, and that might be unrepresentative of its overall diet. While the study of the stomach contents of multiple individuals does mitigate this drawback significantly, it still exists. To account for these issues with stomach content analysis, it is increasingly common to use stable isotope analysis, either on its own (Jackson et al. 2020), or in conjunction with stomach contents analysis (Horswill et al. 2018) to study change in diet. Stable isotope analysis has the added advantage of permitting dietary studies well beyond the timeframe when soft tissues, such as partially digested stomach remains, can be expected to survive. There has already been work done using stable isotope analysis to understand the changing ecology of ichthyofauna in Lake Titicaca (Miller et al. 2010) and the diet of people in the Lake Titicaca region (Miller et al. 2021).

A stable isotope study in grebes would have to focus on changes in the ichthyological element of their diet, both because of the difficulty in identifying invertebrates in the lake in the present and the lack of historic collections of Lake Titicaca invertebrates. Focusing on the ichthyological aspect of their diet would also help answer questions related to how piscivorous grebes partition fish with introduced piscivorous salmonids. Scientists would have to work with local fishermen to get a large enough sample of individuals captured as fisheries bycatch to do this study ethically, while fish could be purchased at market rates from those same fishers. Given that the purpose of such a study would be to look at historic dietary change and hence involve the use of museum specimens, it is likely that grebe specimens would have to be primarily contour feathers, rather than primaries, as have been used in other studies of historic changes in bird diets (Blight et al. 2015). The Titicaca Grebe is flightless, and neither the Silvery Grebe nor Rolland's Grebe is migratory, making the sample of potential prey species relatively restricted, meaning that any researchers studying the diets of these species would not have to worry about their being exposed to any isoscapes alien to Lake Titicaca.

## Author Contributions

**D. A. Villar:** formal analysis (lead), investigation (supporting), methodology (equal), software (lead), visualization (lead), writing – original draft (lead), writing – review and editing (lead). **Ever Yanes:** conceptualization (equal), data curation (lead), investigation (lead), project administration (equal), resources (lead), writing – review and editing (supporting). **Edwin R. Gutiérrez Tito:** data curation (supporting), investigation (supporting), writing – review and editing (equal). **Andrew G. Gosler:** supervision (lead), writing – original draft (equal), writing – review and editing (lead).

## Conflicts of Interest

The authors declare no conflicts of interest.

## Supporting information


**Data S1:** Supporting Information.

## Data Availability

All data used and R code used in this paper is available in the Supporting Information.
